# Dose-Dependent Antagonistic Effects of Montmorillonite on Cadmium Removal Efficiency and Metabolic Defense of *Aspergillus niger*

**DOI:** 10.3390/biology15171564

**Published:** 2026-09-07

**Authors:** Lin Zhang, Yuwen He, Kun Mao, Zhendong Hong, Chen He, Chenhui Wei, Qiaoming Zhang

**Affiliations:** College of Horticulture and Plant Protection, Henan University of Science and Technology, Luoyang 471023, China; zhagln20@126.com (L.Z.); ywhe1003@163.com (Y.H.); 18538361787@163.com (K.M.); chenhehaust@126.com (C.H.); weichenhui1989@163.com (C.W.); zhangqm1013@haust.edu.cn (Q.Z.)

**Keywords:** *Aspergillus niger*, montmorillonite dosage, cadmium removal, metabolic activity

## Abstract

Cadmium (Cd) pollution threatens global ecosystems and human health owing to its high toxicity, long-term persistence, and dual irreversibility of environmental geochemical behavior and biological toxicological effects. The interaction between phosphate-solubilizing fungi (PSF) and clay minerals governs the biogeochemical transformation, chemical speciation, and migration fate of soil Cd. However, the dose-dependent mechanisms underlying Cd^2+^ removal by *Aspergillus niger* (*A. niger*) and montmorillonite remain unclear. In this study, three Cd^2+^ stress levels were simulated, and four *A. niger*-montmorillonite composite systems were established. Key physicochemical and metabolic indicators, including pH, available phosphorus (AP) concentration, oxalic acid concentration, acid phosphatase (ACP) activity, and Cd^2+^ removal rate, were determined. The results showed that the single *A. niger* treatment achieved the optimal Cd removal performance among all composite treatment groups. Severe Cd^2+^ stress induced abundant oxalic acid and ACP secretion, which simultaneously mobilized phosphorus and facilitated Cd removal via phosphate precipitation and oxalate complexation. Low-to-medium montmorillonite dosage temporarily alleviated Cd^2+^ toxicity, whereas high montmorillonite dosage restricted fungal metabolic exudates and, together with acid-mediated mineral-Cd interactions, triggered antagonistic rather than synergistic Cd removal. This study clarifies how montmorillonite dosage reshapes the metabolic defense of *A. niger*, providing theoretical references for optimizing microbe–montmorillonite composite remediation formulations.

## Simple Summary

Cadmium pollution originating from industrial production and agricultural activities has emerged as a critical global environmental threat to ecosystem stability and human health. *Aspergillus niger* can secrete enzymes and organic acids to release phosphate ions, which form stable cadmium-phosphate precipitates, and these organic acids bind cadmium ions, while the fungus employs intracellular antioxidant defense and vacuolar sequestration to alleviate cadmium toxicity. Naturally occurring montmorillonite interacts closely with this functional fungus and collectively regulates cadmium migration and stability in agricultural soil. However, the effect of montmorillonite dosage on fungal-mediated cadmium remediation remains poorly understood. This study confirmed that single fungal treatment achieved a higher cadmium removal rate compared with all fungus-montmorillonite combined systems. Moderate montmorillonite addition alleviated cadmium toxicity and maintained normal fungal activity, while excessive montmorillonite suppressed fungal metabolic resistance and weakened cadmium removal capacity. These findings provide theoretical guidance for developing microbial remediation strategies for cadmium-contaminated soils.

## 1. Introduction

Cadmium (Cd) is a ubiquitous, non-essential potentially toxic element (PTE) that is environmentally persistent and causes severe biological damage even at trace concentrations [1]. Through continuous bioaccumulation throughout food webs, chronic dietary exposure to Cd-enriched crops markedly increases the incidence of osteoporosis in human populations [2,3]. As a priority-controlled inorganic pollutant in China, Cd exhibits a soil over-limit ratio of 7.0%, the highest level among all inorganic soil contaminants [4].

Compared with physical and chemical approaches, microbial remediation is widely regarded as a promising eco-compatible strategy for remediating PTE-polluted soils [5]. Phosphate-solubilizing microorganisms (PSMs) mitigate soil PTE risks by lowering their bioavailability and mobility via multiple pathways, including surface adsorption, ionic complexation, mineral precipitation, and redox transformation [6]. *Aspergillus niger* (*A. niger*), a representative phosphate-solubilizing fungus (PSF), displays prominent remediation merits, including large biomass yield and strong environmental adaptability [7,8]. Stable colonization and proliferation of *A. niger* within soil matrices are an indispensable prerequisite for sustaining its phosphate-solubilizing and Cd-sequestration capacities [9]. However, the strong cytotoxicity of Cd severely suppresses microbial growth, survival, and metabolic activity, which inevitably impairs the remediation performance of single microbial systems and limits their large-scale field application [10]. Our previous research confirmed that *A. niger* possesses significantly higher tolerance to Cd^2+^ stress (up to 50 mg/L) [8]. Benefiting from its multifaceted Cd sequestration pathways, *A. niger* has emerged as a model fungal strain for PTE removal [11].

Clay minerals are among the most abundant and geographically widespread natural components of soils, offering significant advantages in terms of low cost and ready availability [12,13]. Their unique structural properties, including nanoscale particle size, large specific surface area, and a layered porous framework, underpin their effectiveness as adsorbents [14,15]. These favorable characteristics render them cost-effective and sustainable amendments for the in-situ remediation of Cd-contaminated soils [16]. Among clay minerals, montmorillonite stands out due to its markedly higher specific surface area and cation exchange capacity compared to illite and kaolinite, which confer superior adsorptive performance [12,17,18,19,20]. These structural merits make montmorillonite a particularly attractive candidate for the stabilization of PTEs, and it has thus garnered considerable research interest in contaminated soil remediation. However, sole montmorillonite amendment achieves transient Cd removal rather than permanent Cd elimination. Immobilized Cd can readily undergo re-mobilization in response to dynamic fluctuations in soil pH, redox potential and organic matter concentration. This fundamental limitation provides a clear theoretical rationale for developing integrated systems combining mineral protection and microbial remediation.

The coexistence and interaction between PSM and clay minerals create a critical micro-interface that connects soil biota to soil minerals [12,21], thereby governing the speciation, stability and mobility of soil PTEs [22]. Clay minerals provide stable ecological niches for fungal colonization and growth, effectively improving fungal adaptability and stress resistance under PTE contamination [23]. Conversely, fungal metabolism can further promote mineral precipitation, dissolution, and structural transformation by modifying soil microenvironmental conditions, thereby facilitating biomineralization processes [22,23]. Composite aggregates and secondary metabolites generated via fungus-clay mineral interactions introduce abundant additional adsorption sites, which collaboratively stabilize soil Cd through synergistic adsorption, complexation, and co-precipitation [24]. Hence, the fungus–montmorillonite composite system represents an environmentally sustainable alternative strategy for remediation. The efficient adsorption of Cd^2+^ by montmorillonite can substantially reduce the concentration of free Cd^2+^, thereby alleviating Cd-induced toxicity to *A. niger* and preserving the fungus’s metabolic activity [25]. Previous studies have explored the remediation potential of microbe–clay mineral composites, yet most published literature centers on bacteria–mineral binary systems [24,26]. Notably, PTE adsorption and immobilization by bacteria–mineral composites are highly condition-dependent. Their adsorption performance, metal speciation transformation, and adsorption capacity are directly affected by intrinsic factors, including microbial dosage, mineral type, microorganism-mineral combination mode and mass ratio, and are also regulated by environmental factors such as pH and ionic strength [27,28]. In contrast, systematic investigations targeting *A. niger*–montmorillonite composite remediation remain scarce.

Given the complexity of actual Cd-contaminated soil environments involving indigenous microorganisms, organic matter, and multi-mineral competitive interference, this study systematically investigated the dose-dependent interaction mechanisms between *A. niger* and montmorillonite under controlled liquid conditions. A series of indicators was determined, including pH values, available phosphorus (AP) concentrations, low-molecular-weight organic acid concentrations, acid phosphatase (ACP) activity, residual Cd^2+^ concentration, and Cd^2+^ removal rate. Meanwhile, the morphological characteristics and interfacial interaction mechanisms of *A. niger* and montmorillonite were analyzed. We hypothesized that (1) antagonistic rather than synergistic effects on Cd removal exist in the *A. niger*–montmorillonite composite system, (2) montmorillonite exerts dose-dependent regulatory effects on the Cd removal performance of the co-culture system, and (3) the antagonistic interactions are jointly driven by physical encapsulation of fungal hyphae and inhibition of fungal metabolism.

## 2. Materials and Methods

### 2.1. Sample Preparation

The fungal strain used in this study was *A. niger* (CGMCC No.11544) strain NJDL-12, which was previously isolated from the rhizosphere soil of soybean in Nanjing, China [29]. Growth and sporulation of *A. niger* were achieved by culturing it on potato dextrose agar (PDA) for seven days at 28 °C. Analytical grade Cd nitrate tetrahydrate (98%, Cd(NO_3_)_2_4H_2_O) (Sigma-Aldrich, St. Louis, MO, USA) was used. The clay mineral used was sodium-based montmorillonite (SWy-2), purchased from the Clay Mineral Society (Purdue University, West Lafayette, IN, USA), which was studied in detail [30].

### 2.2. Fungal-Mineral Co-Culture Experiments Under Gradient Cd^2+^ Stress

A liquid shake-flask experiment was performed to explore the Cd removal potential of the combined *A. niger*-montmorillonite system across gradient montmorillonite dosages and Cd^2+^ stress levels. Based on our previous experimental results [30], four montmorillonite dosage treatments were set, including Mon0 (blank control without montmorillonite), Mon1 (10 mg montmorillonite), Mon2 (30 mg montmorillonite), and Mon3 (50 mg montmorillonite). Three Cd contamination gradients were also configured by adding Cd stock solution (1000 mg/L Cd^2+^), namely Cd0 (control, 0 mg/L Cd^2+^), Cd1 (2 mg/L Cd^2+^), and Cd2 (4 mg/L Cd^2+^). Briefly, 100 mL PDA medium was dispensed into 250 mL Erlenmeyer flasks and autoclaved at 115 °C for 30 min. Then, each flask was inoculated with 1 mL of *A. niger* spore suspension (ANG, ~1.36 × 10^6^ spores/mL), followed by the addition of the corresponding pre-set montmorillonite dosage and Cd stock solution according to the experimental design (Table 1). The incubation was performed at 28 °C for 7 days in the dark with shaking at 180 rpm. All the treatments had four replicates.

After 7 days of incubation, the fungal-mineral aggregates were separated from the medium through filter paper (Cytiva Biotechnology Co., Ltd., Hangzhou, China) and repeatedly rinsed with sterile Milli-Q water at least three times to remove residual medium and free cadmium ions on the aggregate surface. Subsequently, partial fungal-mineral aggregates were fixed, dehydrated, and gold-sputtered in preparation for scanning electron microscopy (SEM) observation and energy-dispersive X-ray spectroscopy (EDS) elemental mapping to analyze morphological characteristics and interfacial elemental distribution. Meanwhile, the collected culture supernatants were centrifuged at 2553 g for 30 min to remove residual mycelial fragments and mineral precipitates and filtered through a 0.22 µm pore size polyether sulfone membrane ( Keyilong Lab Equipment Co., Ltd., Tianjin, China). Finally, the prepared supernatant samples were used for the determination of pH value, residual Cd^2+^ concentration, oxalic acid concentration, ACP activity, and AP concentration.

### 2.3. Microstructure and Morphology of Fungal-Mineral Interaction

The surface morphology and micro-area chemical composition of the samples were investigated using a Carl Zeiss EVO10 tungsten filament scanning electron microscope (Carl Zeiss AG, Oberkochen, Germany). An Oxford Xplore 30 energy-dispersive X-ray spectrometer (Oxford Instruments NanoAnalysis, Abingdon, UK) was coupled to the SEM for elemental composition analysis. To suppress charging effects and obtain high-quality micrographs, all specimens were sputter-coated with gold for 60 s before testing. An accelerating voltage of 15 kV was used for both SEM morphological observation and EDS elemental detection.

### 2.4. Chemical Properties of the Medium

The medium pH values were measured using a pH meter (LeiMing PHS-3C, Shanghai, China). AP concentrations were determined via inductively coupled plasma optical emission spectrometry (Agilent 710 ICP-OES, Agilent Technologies, Santa Clara, CA, USA).

### 2.5. Fungal Metabolic Activity Analysis

The oxalic acid concentrations were quantified using a high-performance liquid chromatography (HPLC) system (Agilent 1260 Infinity II). The chromatographic detection conditions were set as follows: the mobile phase consisted of a mixture of 0.25% potassium dihydrogen phosphate solution and methanol at a volume ratio of 99:1. An Agilent Zorbax SB-Aq chromatographic column was used, with the column temperature maintained at 30 °C. The injection volume, flow rate and detection wavelength were set as 20 μL, 0.8 mL/min and 210 nm, respectively. A series of oxalic acid standard solutions with concentrations ranging from 0 to 1000 mg/L were prepared for standard curve calibration. The calibrated curve exhibited excellent linearity, with a coefficient of determination (R^2^) of 0.999, which satisfied the quantitative detection requirements. The acid phosphatase (ACP) activity was determined using a commercial biochemical assay kit (No. A060-1-1, Nanjing Jiancheng Biotechnology Co., Ltd., Nanjing, China) in accordance with the manufacturer’s protocols. Specifically, the culture solutions were first filtered through qualitative filter paper and further purified with a 0.45 μm microporous membrane to obtain transparent supernatant. The ACP activities were calculated based on Equation (1):

ACP (U/100 mL) = A_d_/A_s_ × m_s_ × (100/V_s_), (1)

where m_s_ is the mass of the standard substance (0.005 mg), V_s_ is the actual sampling volume (0.05 mL), A_d_ denotes the average absorbance values of sample groups, and A_s_ denotes that of standard groups.

### 2.6. Determination of Cd^2+^ Removal Rate

After 7 days of cultivation, the culture solutions were filtered to separate fungal mycelia and supernatant. The filtrates were then passed through a 0.45 μm membrane, and the residual Cd^2+^ concentrations were determined by flame atomic absorption spectrometry Agilent 200 Series FAAS. Cd^2+^ was measured at a hollow cathode lamp wavelength of 228.8 nm. The standard working curve covered a linear range from 0 to 5 mg/L with a coefficient of determination (R^2^) of 0.9997. The instrument limit of detection (LOD) was 0.003 mg, and the limit of quantification (LOQ) was 0.010 mg. Blank medium calibration and standard reference material recovery tests were conducted for quality control, with cadmium recovery rates ranging from 94.2% to 105.1%, verifying the accuracy of the detection system. The Cd^2+^ removal rate of each treatment was calculated using Equation (2):

Removal rate of Cd (%) = (C_im_ − C_fm_)/C_im_ × 100%, (2)

where C_im_ and C_fm_ represent the initial and final Cd^2+^ concentrations (mg/L) in the medium, respectively.

### 2.7. Statistical Analysis

Two-way analysis of variance (ANOVA) was performedusing SPSS 26.0 to quantify the main effects of montmorillonite addition, the main effects of Cd^2+^concentration, and their interactive effects on all response indicators (pH value, AP concentration, oxalic acid concentration, ACP activity, residual soluble Cd^2+^ concentration, and Cd^2+^ removal efficiency). Post-hoc Tukey multiple comparisons were conducted to identify significant differences among individual treatments at *p* < 0.05.

## 3. Results

### 3.1. Morphological Investigation

**Figure 1:**
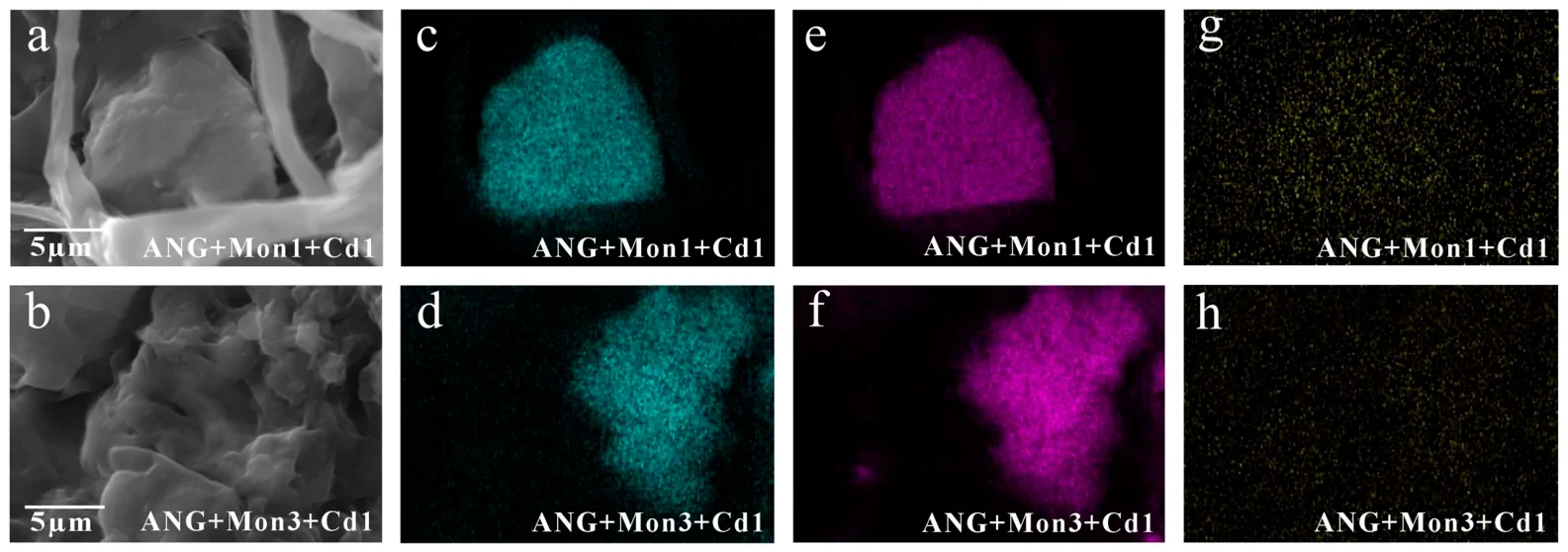
SEM images of *A. niger* co-cultured with Mon1 (**a**) and Mon3 (**b**) after 7 days of incubation (scale bar represents 5 μm). EDS showed the chemical distribution of Al (**c**,**d**), Si (**e**,**f**), and Cd (**g**,**h**) in the presence of different amounts of Mon.

Microscopic examination elucidated changes in mineral and fungal morphology after seven days of incubation (Figure 1). SEM analysis showed that the hyphae of *A. niger* surrounded relatively large montmorillonite particles (<10 μm) in the ANG + Mon1 + Cd1 and ANG + Mon3 + Cd1 treatments (Figure 1a,b), which were also confirmed by the enriched aluminum (Al) and silicon (Si) signals in EDS mapping (Figure 1c–f). EDS mapping revealed intense Cd signals distributed in the ANG + Mon1 + Cd1 treatment (Figure 1g). In contrast, relatively weak Cd signals were detected within the mycelia after Mon3 addition (Figure 1h).

### 3.2. pH Values and AP Concentrations

**Figure 2:**
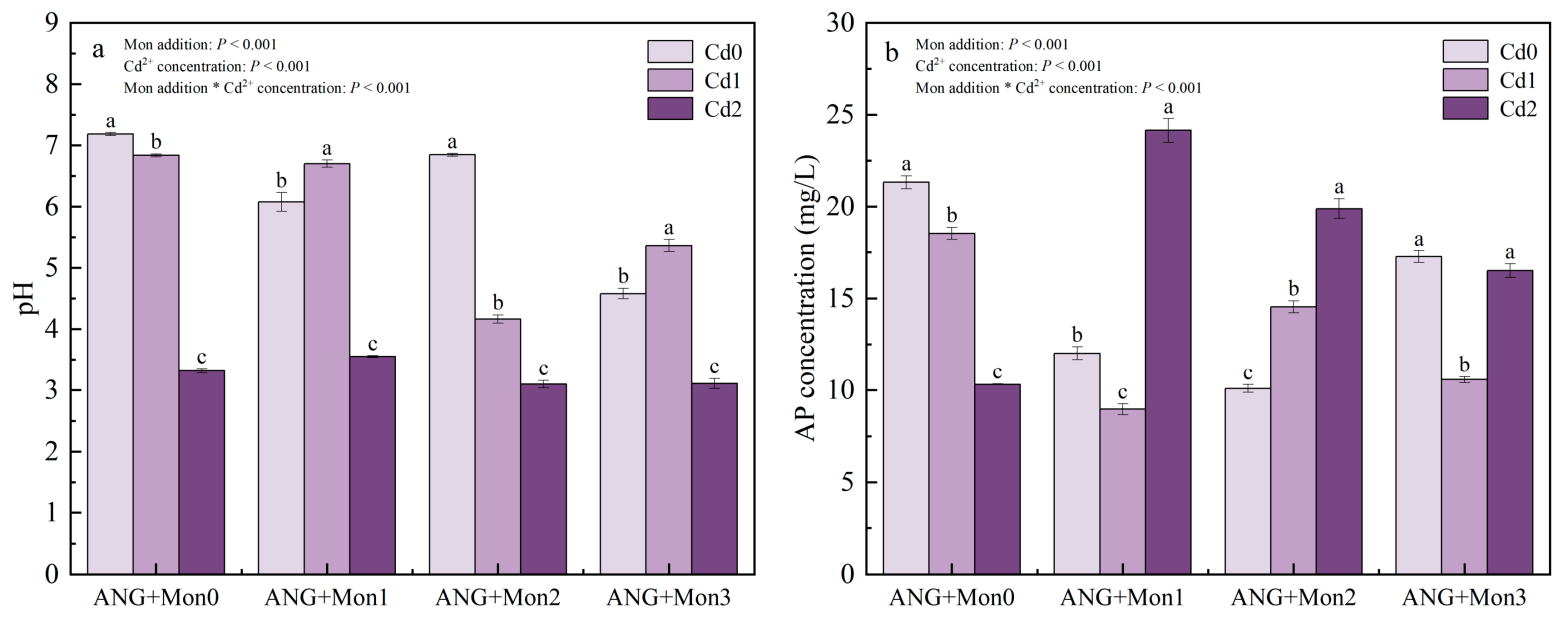
Acidity (**a**) and AP concentrations (**b**) in the medium after 7 days of incubation. Different lowercase letters indicate significant differences among Cd^2+^ treatments within the same Mon addition levels (p < 0.05). Asterisks (*) indicate interactions.

Changes in pH values of medium in the fungal-montmorillonite composite system after 7 days of incubation are presented in Figure 2a. For the ANG + Mon0 treatment, the pH value of the medium gradually decreased as Cd^2+^ concentration increased, with the most pronounced reduction observed under the highest Cd^2+^ stress (Cd2), where the pH dropped to 3.32 (*p <* 0.05). In the ANG + Mon1 treatment, the pH value first increased from 6.07 to 6.70 and then decreased to 3.55 (*p <* 0.05), exhibiting a trend of an initial rise followed by a decline. A continuous and significant pH reduction was detected throughout the ANG + Mon2 treatment. Similar to the pattern observed in the ANG + Mon1 treatment, the ANG + Mon3 treatment presented a pH elevation from 4.57 to 5.36, followed by a sharp decline to 3.11 (*p <* 0.05), which was consistent with the trend observed in the ANG + Mon1 treatment. Overall, the Cd2 treatment induced the lowest pH values across all fungal-mineral composite treatments.

Figure 2b showed the variation of AP concentrations in the medium under different treatments. In the ANG + Mon0 treatment, AP concentration decreased with the elevation of Cd^2+^ levels, demonstrating that Cd^2+^ stress significantly inhibited the growth and metabolic activity of *A. niger*. In the ANG + Mon1 treatment, AP concentration exhibited a distinct trend of initial decline followed by a subsequent increase, with values of 12.01 mg/L (Cd0), 8.96 mg/L (Cd1), and 24.14 mg/L (Cd2) (*p* < 0.05). In the ANG + Mon2 treatment, AP concentrations showed a continuously significant upward trend, which were 10.09 mg/L (Cd0), 14.54 mg/L (Cd1), and 19.89 mg/L (Cd2) (*p <* 0.05). AP concentrations initially decreased and then increased in the ANG + Mon3 treatment, implying that high-dose montmorillonite (Mon3) facilitated P activation but exhibited unstable P release performance under Cd^2+^ stress. Among the ANG + Mon1, ANG + Mon2, and ANG + Mon3 treatments, the maximum AP concentrations were detected under Cd2 exposure. At the Cd2 contamination level, AP concentration decreased with rising montmorillonite dosage, suggesting that montmorillonite dosage exerted a prominent interference effect on P adsorption and release.

### 3.3. Oxalic Acid Concentration and ACP Activity

**Figure 3:**
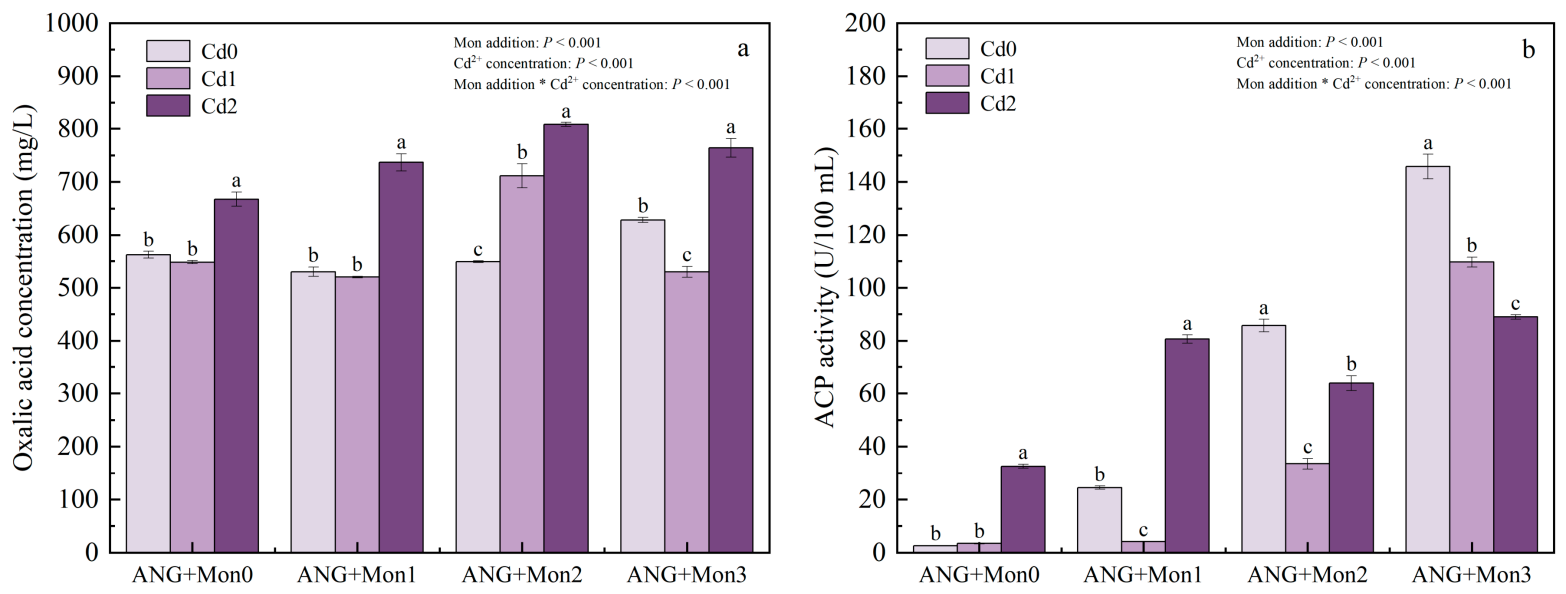
Oxalic acid concentrations (**a**) and ACP activity (**b**) in the medium with different combined treatments of *A. niger* and montmorillonite. Different lowercase letters indicate significant differences among Cd^2+^ treatments within the same Mon addition levels (*p* < 0.05). Asterisks (*) indicate interactions.

Oxalic acid has been identified as the primary secretion of *A. niger*. The variations in oxalic acid secretion under all treatments are shown in Figure 3a. For the ANG + Mon0 treatment, oxalic acid concentrations under Cd0 and Cd1 were 562.34 and 548.07 mg/L, respectively, with no significant difference between the two treatments. By contrast, the oxalic acid concentration under Cd2 reached 667.50 mg/L (*p <* 0.05), demonstrating that high Cd^2+^ stress (Cd2) stimulated substantial oxalic acid excretion by the strain. In the ANG + Mon1 treatment, oxalic acid levels were 530.07 mg/L (Cd0) and 520.11 mg/L (Cd1) with non-significant variation, whereas the value rose to 736.66 mg/L under Cd2 (*p <* 0.05). In the ANG + Mon2 treatment, oxalic acid production increased along with elevated Cd exposure intensity. In the ANG + Mon3 treatment, oxalic acid concentration displayed a trend of initial decline followed by elevation, which implied that the ANG + Mon3 combination suppressed oxalic acid secretion under moderate Cd1 stress. Overall, oxalic acid secretion was markedly enhanced as Cd^2+^ concentration increased, and all Cd2 treatments produced greater oxalic acid amounts than Cd0 and Cd1 treatments.

The supplementation of montmorillonite and varying Cd^2+^ concentrations exerted significant effects on the ACP activity (Figure 3b). In the ANG + Mon0 treatment, ACP activity remained low and showed no statistically significant difference between Cd0 (2.60 U/100 mL) and Cd1 (3.44 U/100 mL). In contrast, enzyme activity increased drastically to 32.60 U/100 mL under Cd2 stress (*p <* 0.05). This result indicated that severe Cd^2+^ stress (Cd2) triggered elevated ACP production as a stress defensive response. Both ANG + Mon1 and ANG + Mon2 treatments showed a universal pattern of initial reduction and subsequent elevation in ACP activity. This variation suggested that low-dose montmorillonite (Mon1) mitigated Cd toxicity at a moderate Cd^2+^ level (2 mg/L), which temporarily lowered ACP activity. Nevertheless, the stress-mitigating capacity of Mon1 failed to function under high Cd^2+^ stress (4 mg/L), aggravating metal toxicity and further boosting ACP activity. Conversely, ACP activity declined linearly with significant differences across the Cd gradient in the ANG + Mon3 treatment. High-dose montmorillonite supplementation (50 mg) induced remarkably high ACP activity under Cd-free conditions; however, enzyme activity decreased progressively as Cd stress intensified.

### 3.4. Residual Cd^2+^ Concentration and Cd^2+^ Removal Rate

**Figure 4:**
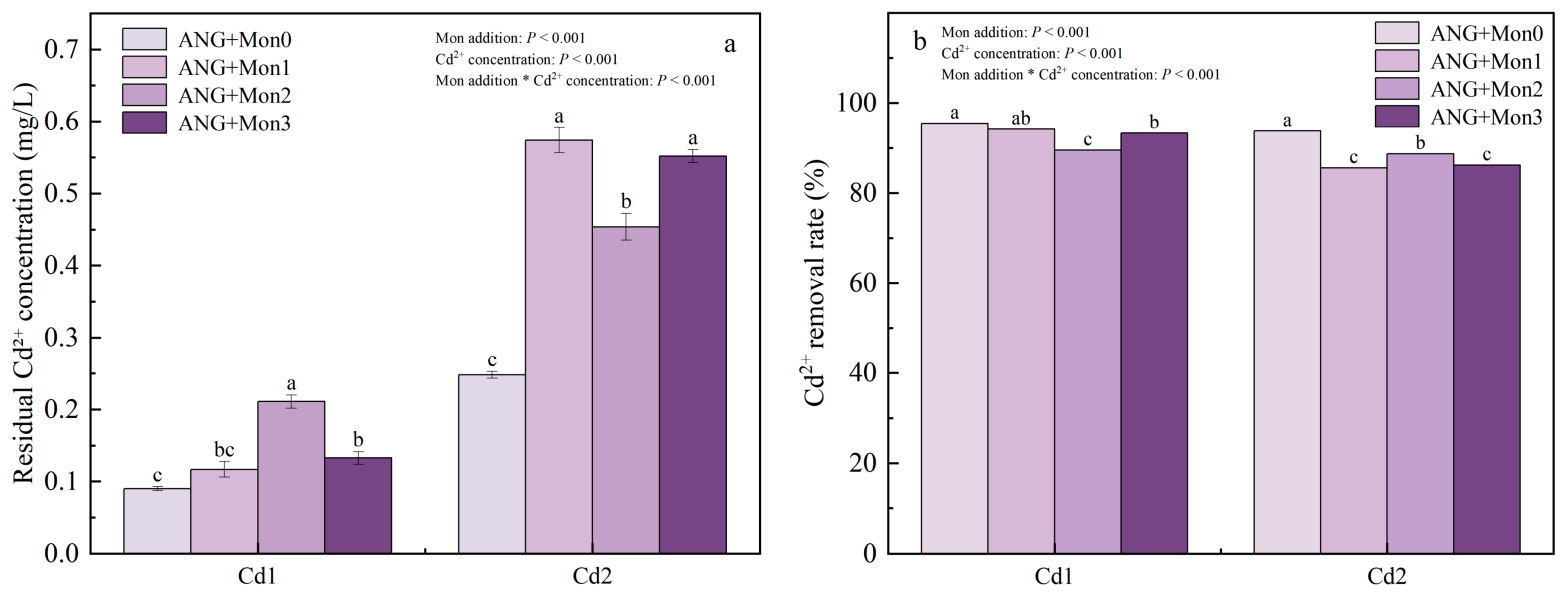
Residual Cd^2+^ concentrations (**a**) and Cd^2+^ removal rates (**b**) in the medium with different combined treatments of *A. niger* and montmorillonite. Different lowercase letters indicate significant differences among Cd^2+^ treatments within the same Mon addition levels (*p* < 0.05). Asterisks (*) indicate interactions.

Residual Cd^2+^ concentrations in all treatments were markedly higher under high Cd2 stress than under moderate Cd1 stress, regardless of montmorillonite addition (Figure 4a). Under Cd1 exposure, residual Cd^2+^ levels first increased and then declined as montmorillonite dosage increased. The ANG + Mon2 treatment exhibited the highest residual Cd^2+^ concentration at 0.211 mg/L, which was significantly greater than those of ANG + Mon0 (0.090 mg/L) and ANG + Mon3 (0.133 mg/L) (*p <* 0.05). The ANG + Mon1 treatment presented an intermediate residual Cd^2+^ concentration of 0.117 mg/L, with no significant differences relative to ANG + Mon0 or ANG + Mon3. By contrast, ANG + Mon0 possessed the minimum residual Cd^2+^ concentration when subjected to Cd2 stress. Markedly higher residual Cd^2+^ contents were detected in both ANG + Mon1 and ANG + Mon3, and no significant distinction existed between ANG + Mon1 (0.574 mg/L) and ANG + Mon2 (0.454 mg/L). Overall, the single *A. niger* treatment (ANG + Mon0) sustained the lowest residual Cd^2+^ concentration at both Cd contamination levels. Supplementing montmorillonite generally increased residual soluble Cd^2+^ in the culture medium, revealing that the *A. niger*–montmorillonite composite systems failed to further immobilize Cd^2+^ effectively compared with pure fungal incubation.

The Cd^2+^ removal rate of all *A. niger*–montmorillonite co-culture treatments exceeded 85% under both moderate Cd1 and severe Cd2 stress (Figure 4b). Under Cd1 exposure, the ANG + Mon0 treatment achieved the maximum Cd^2+^ removal rate of 95.52%, which was significantly higher than the ANG + Mon2 treatment (89.45%). No statistically significant difference was observed between ANG + Mon1 treatment and ANG + Mon0 treatment, whereas ANG + Mon3 treatment exhibited markedly higher Cd removal performance than the ANG + Mon2 treatments. When subjected to Cd2 stress, ANG + Mon0 treatment still maintained the optimal Cd^2+^ removal rate of 93.81%. Then, Cd^2+^ removal rate dropped sharply to 85.64% in the ANG + Mon1 treatment, rebounded moderately to 88.66% in the ANG + Mon2 treatment, and decreased significantly to 86.20% in the ANG + Mon3 treatment compared with the ANG + Mon2 treatment. This finding suggests that montmorillonite supplementation failed to continuously promote Cd remediation performance.

## 4. Discussion

*A. niger* sequesters Cd and alleviates its toxicity via multiple extracellular and intracellular pathways. Under Cd stress of 2 mg/L and 4 mg/L, only *A. niger* addition exhibited the highest Cd^2+^ removal efficiency (Figure 4), which was consistent with our previous findings [8]. The underlying mechanisms are described as follows. Firstly, components of the fungal cell wall play an important role in metal binding [8]. Additionally, oxalic acid is a primary low-molecular-weight organic acid secreted by *A. niger*, which contributes to metal detoxification through the formation of stable oxalate complexes [9,10]. Beyond organic acid production, *A. niger* also secretes phosphatases that mineralize organic phosphorus compounds, releasing phosphate ions that can precipitate toxic metals [31,32]. Upon entering fungal cells, metal cations can be sequestered and/or precipitated within various cellular organelles while intracellular antioxidant defense systems are activated to counteract Cd-elicited oxidative stress, reduce cytosolic Cd bioavailability, and enhance metal tolerance and remediation performance [33,34].

Interactions between microorganisms and clay minerals are ubiquitous in nature [35]. These relations drive global biogeochemical cycles and element transformation phenomena [33]. SEM-EDS observations revealed tight physical adhesion between *A. niger* hyphae and montmorillonite particles to form microbe-mineral aggregates serving as protective microhabitats against Cd toxicity (Figure 1). Distinct Cd distribution patterns were observed across montmorillonite gradient treatments: low-dose Mon1 treatment showed concentrated Cd signals surrounding hyphae (Cd^2+^ accumulates on fungal surfaces), while high-dose Mon3 treatment displayed weak Cd signals around hyphae (mineral coating isolates fungi from free Cd^2+^), indicating that excessive montmorillonite disrupted Cd sequestration at the fungus-mineral interface. Such gradient-dependent interfacial differences reveal that montmorillonite dosage critically modulates fungus-mediated Cd^2+^ retention behavior, with obvious divergence between low and high mineral supplementation.

**Figure 5:**
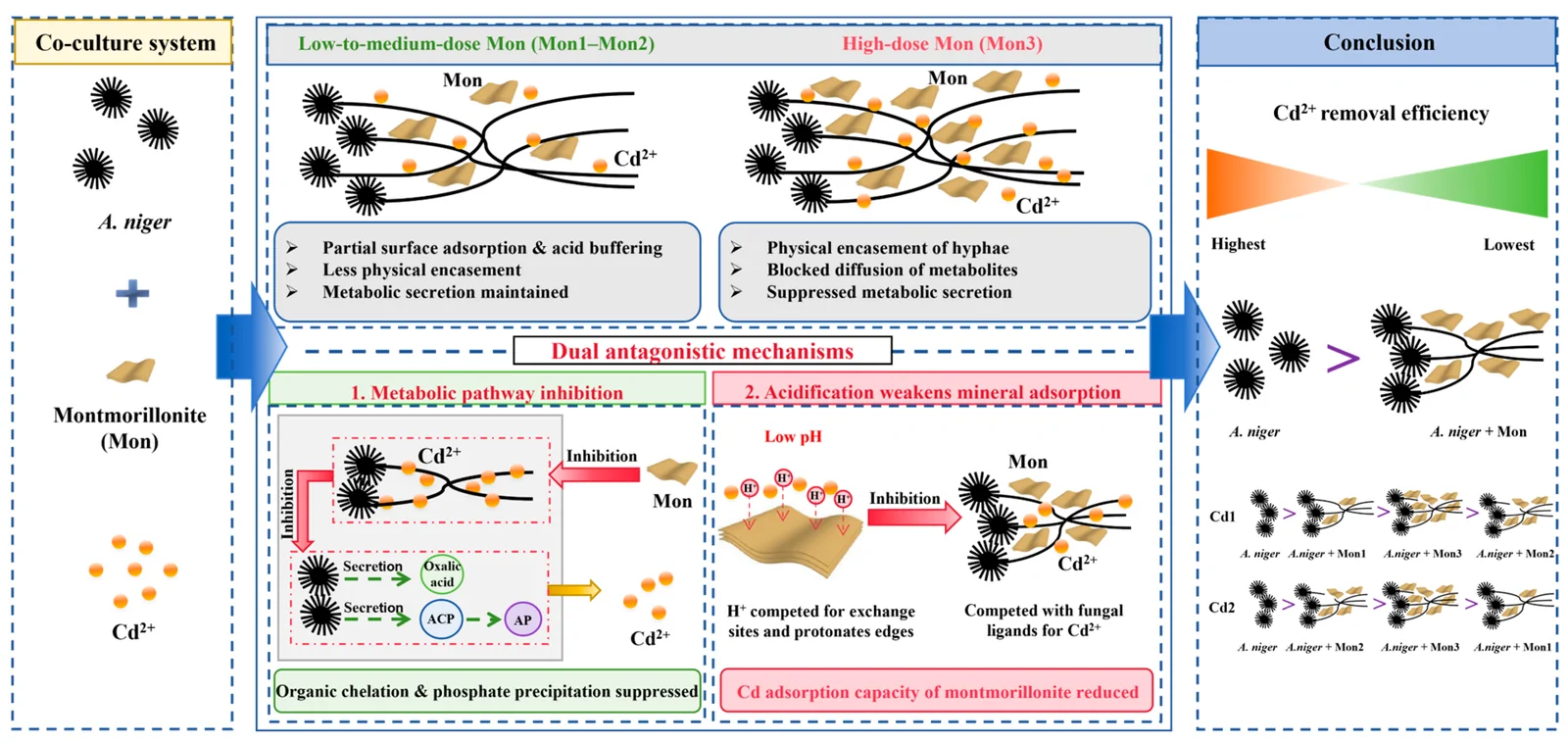
Schematic illustration of dose-dependent antagonistic mechanisms of montmorillonite suppressing Cd removal and metabolic secretion of *A. niger*.

The divergent Cd distribution characteristics observed under the montmorillonite gradient at the microscale were further reflected in the overall Cd removal performance of the liquid co-culture system. All treatments achieved Cd^2+^ removal rates over 85% at initial Cd^2+^ concentrations lower than 4 mg/L, confirming the strong inherent Cd sequestration capacity of *A. niger* alone [8,36,37]. As a typical 2:1 layered phyllosilicate, montmorillonite possesses inherent structural advantages including high cation exchange capacity, large specific surface area and permanent negative surface charges originating from isomorphic substitution, which endow it with natural Cd^2+^ sequestration potential via ion exchange and outer-sphere surface complexation [15,38,39]. Although both *A. niger* and montmorillonite individually possess considerable Cd^2+^ removal capacity [8,40], montmorillonite addition failed to improve remediation performance in the co-culture system, and obvious antagonistic effects rather than anticipated synergies were observed. The single *A. niger* treatment maintained the lowest soluble residual Cd^2+^ and highest removal rate under both mild and severe Cd stress, whereas elevated montmorillonite dosage consistently increased dissolved Cd^2+^ in the medium and reduced overall Cd^2+^ removal capacity. Two core mechanisms accounted for this antagonistic relationship, as schematically illustrated in Figure 5. The first mechanism refers to physical suppression of fungal metabolic secretion. Oxalic acid and ACP are key metabolites for *A. niger* to achieve metal detoxification and P activation [8,41]. Severe Cd^2+^ stress significantly upregulated secretion of both substances as inherent fungal tolerance strategies, whereas elevated montmorillonite dosages resulted in reduced oxalic acid concentration and ACP activity (Figure 3a,b). Excessive montmorillonite physically encased fungal hyphae and hindered the outward diffusion of oxalic acid and extracellular ACP [30], thereby compromising two dominant Cd fixation pathways: organic chelation and phosphate biomineral co-precipitation [36]. Meanwhile, montmorillonite surfaces competed with fungal organic exudates for phosphate ions, disrupting medium P mobilization balance [42]. Beyond the direct metabolic suppression of fungal strains, chemical interactions between montmorillonite and Cd under acidic conditions further aggravated such antagonism. Cd-induced severe acidification significantly weakened the Cd adsorption capacity of montmorillonite [43,44]. The surface charge properties of montmorillonite are highly pH-sensitive [43]; massive H^+^ compete for permanent cation exchange sites and protonate variable edge hydroxyl groups under acidic conditions, sharply reducing the mineral’s Cd binding affinity. Moreover, montmorillonite competed with fungal organic ligands for free Cd^2+^ and formed acid-labile mineral-Cd complexes that readily re-dissolved under acidic environments [44,45]. Such competitive binding further suppressed fungal tolerance to Cd stress [35], which directly explained the attenuated Cd enrichment signals in microscopic elemental mapping.

Although low montmorillonite dosage could temporarily alleviate Cd toxicity via surface adsorption and acid buffering relying on its abundant exchangeable cations, metabolic inhibition induced by high mineral addition occupied a dominant position under high Cd or high mineral contamination, ultimately lowering overall Cd removal efficiency. Consistent with the above dual antagonistic mechanisms, this liquid incubation experiment clearly demonstrated that montmorillonite cannot synergistically boost the Cd remediation performance of *A. niger*, and pure fungal inoculation exhibited superior Cd fixation capacity under liquid culture conditions. The dose-dependent antagonistic interaction identified in this study provides clear guidance for optimizing microbe-mineral combined remediation formulas: simply mixing clay minerals with PSF cannot guarantee stable Cd removal, and mineral addition amounts must be matched to microbial metabolic characteristics to avoid metabolic inhibition.

Notably, all conclusions are drawn from simplified liquid media without complex interference factors in natural soil, including indigenous microorganisms, soil organic matter, and multi-mineral composite matrices [46]. These factors have been proven to significantly regulate microbial physiological activity, as well as Cd removal and transformation efficiency in soil [21,47]. This study aims to disentangle the independent interaction between *A. niger* and montmorillonite. A serial montmorillonite-addition gradient ranging from 0 to 50 mg was set up to identify the critical threshold dosage triggering metabolic antagonism in this fungal strain. The mechanistic evidence in this study serves as fundamental data for subsequent heterogeneous soil simulation experiments, rather than replicating the intrinsic mineral background concentrations of natural agricultural soils. Although montmorillonite is naturally non-limiting in most soils, coexisting humus and Fe/Al oxides will coat clay surfaces and weaken hyphal encapsulation, meaning the severe antagonism seen in liquid may not fully emerge in intact bulk soil. We have marked this systematic gap between liquid simulation and real soil as a key limitation of this study. From an engineering perspective, our gradient results deliver practical guidance: current field remediation widely combines exogenous montmorillonite passivators with PSF, but dosage matching is rarely optimized. Insufficient clay cannot mitigate fungal Cd toxicity, while over-dosing suppresses detoxification metabolism and causes material waste. This study quantifies the tolerable upper limit of montmorillonite for *A. niger*, providing clear references for remediation formula design. Follow-up soil microcosm incubation and field-scale trials integrated with sequential chemical fractionation assays are required to quantify differential cadmium geochemical fractions and validate whether the observed dose-dependent antagonistic interaction persists in natural Cd-contaminated soil matrices. Future research can also develop targeted regulatory approaches to mitigate the metabolic suppression triggered by overdosed montmorillonite, thereby exploring viable technical pathways to achieve high-efficiency synergistic Cd remediation via fungus–mineral composite systems under authentic soil conditions.

## 5. Conclusions

This study systematically quantified medium physicochemical properties, fungal metabolic characteristics, soluble residual Cd^2+^ concentrations and Cd^2+^ removal rate in the *A. niger*-montmorillonite co-culture system under four montmorillonite dosage gradients and three Cd^2+^ contamination levels. The results verified that varying montmorillonite dosages remarkably altered fungal metabolic behaviors and Cd removal performance under consistent incubation conditions, which was mainly mediated by medium acidification, oxalic acid secretion, and acid phosphatase (ACP) activity. The single *A. niger* treatment presented the lowest soluble residual Cd^2+^ and the highest Cd removal rates under both Cd stress levels, benefiting from the intrinsic metabolic detoxification and Cd sequestration capacity of this strain. Furthermore, detection of metabolic indicators revealed elevated biosynthesis of oxalic acid and ACP under high Cd exposure, implying putative robust metal detoxification and phosphorus activation capacity of the strain against severe Cd toxicity. Low and medium montmorillonite dosages (Mon1, Mon2) maintained steady phosphorus mobilization and organic acid production, accompanied by moderate alleviation of Cd-induced growth inhibition, which could hypothetically assist fungi in adapting to Cd stress. High-dose montmorillonite (Mon3) showed weakened Cd removal effects despite stable fungal survival. It tended to suppress the secretion of oxalic acid and ACP and continuously elevated soluble Cd^2+^ concentrations in the medium. Increased montmorillonite dosage may potentially disrupt fungal Cd sequestration pathways by interfering with metabolic exudate production, as reflected by elevated dissolved residual Cd^2+^ and decreased Cd removal rate. Overall, montmorillonite dosage exerts a prominent antagonistic effect on *A. niger*-mediated Cd removal. Increased clay dosage progressively suppresses fungal metabolic activity and weakens the remediation performance of the co-culture system. These exploratory laboratory-scale observations derived solely from simplified liquid co-culture systems merely provide preliminary theoretical references for optimizing microbe–montmorillonite composite remediation formulas under controlled lab conditions and cannot be directly extrapolated to complex field remediation scenarios. Future studies should carry out soil microcosm incubation and field plot experiments to clarify the interfering effects of indigenous microorganisms, soil organic matter and inter-mineral competitive adsorption on fungus–montmorillonite interfacial interactions, to deepen mechanistic insights and lay a solid foundation for developing practical in-situ remediation technologies for Cd-contaminated agricultural soils.

## Figures and Tables

**Table 1:** Experimental design for the combined Cd removal mediated by *A. niger* and Mon addition.

Treatments	Fungal Spore Suspension (mL)	Montmorillonite Addition (mg)	Cd^2+^ Concentration (mg/L)
ANG + Mon0 + Cd0	1	0	0
ANG + Mon0 + Cd1	1	0	2
ANG + Mon0 + Cd2	1	0	4
ANG + Mon1 + Cd0	1	10	0
ANG + Mon1 + Cd1	1	10	2
ANG + Mon1 + Cd2	1	10	4
ANG + Mon2 + Cd0	1	30	0
ANG + Mon2 + Cd1	1	30	2
ANG + Mon2 + Cd2	1	30	4
ANG + Mon3 + Cd0	1	50	0
ANG + Mon3 + Cd1	1	50	2
ANG + Mon3 + Cd2	1	50	4

## Data Availability

The original contributions presented in this study are included in the article. Further inquiries can be directed to the corresponding author.
